# Decellularized tissues as platforms for digestive system cancer models

**DOI:** 10.1016/j.heliyon.2024.e31589

**Published:** 2024-05-21

**Authors:** Zahra Seifi, Mozafar Khazaei, Danial Cheraghali, Leila Rezakhani

**Affiliations:** a Student Research Committee, Kermanshah University of Medical Sciences, Kermanshah, Iran; b Fertility and Infertility Research Center, Health Technology Institute, Kermanshah University of Medical Sciences, Kermanshah, Iran; c Department of Tissue Engineering, School of Medicine, Kermanshah University of Medical Sciences, Kermanshah, Iran; d Department of Mechanical Engineering, New Jersey Institute of Technology, NJ, USA

**Keywords:** Decellularized tissues, Digestive system, Extracellular matrix, Cancer

## Abstract

The extracellular matrix (ECM) is a multifunctional network of macromolecules that regulate various cellular functions and physically support the tissues. Besides physiological conditions, the ECM also changes during pathological conditions such as cancer. As tumor cells proliferate, notable changes occur in the quantity and makeup of the surrounding ECM. Therefore, the role of this noncellular component of tissues in studies of tumor microenvironments should be considered. So far, many attempts have been made to create 2-dimensional (2D) or 3-dimensional (3D) models that can replicate the intricate connections within the tumor microenvironment. Decellularized tissues are proper scaffolds that imitate the complex nature of native ECM. This review aims to summarize 3D models of digestive system cancers based on decellularized ECMs. These ECM-based scaffolds will enable us to study the interactive communication between cells and their surrounding environment which brings new potential for a better understanding of the pathophysiology of cancer.

## 1 Introduction

In recent years, there has been a surge of interest in studying the extracellular matrix (ECM) due to its complex network of macromolecules, which goes beyond a mere stable and physical structure that supports tissue growth [[1], [2], [3]]. Tumor tissues undergo significant remodeling of the ECM, resulting in the loss of structural organization and an overall increase in stiffness, in addition to modification of cancer-related biological processes including invasion, metastasis, angiogenesis, cancer cell immune evasion, stemness, and cell proliferation [[4], [5], [6]]. Alterations in the tumor microenvironment ECM have been linked to the accelerated development of disease and poor prognosis [7]. This highlights the importance of considering this non-cellular element when examining interactions within the tumor microenvironment and creating an innovative therapeutic approach [4,8].

Although most cancer research has been conducted using *in vitro* 2D cell cultures, these models do not possess the 3D cellular organization, cell interactions, and the suitable ECM-supporting microenvironment observed *in vivo* [4,9]. To address these limitations, researchers are increasingly creating 3D culture systems. The utilization of 3D culture can serve as a valuable tool for a better understanding of alterations, interactions, and cellular and molecular signaling during the process of malignant transformation. Culture systems can be either scaffold-based which utilizes natural or artificial solid scaffolds or scaffold-free, such as spheroids that do not rely on scaffolds [10,11].

Several reports have detailed interesting approaches that involve utilizing decellularized ECM from natural tissues, where the cellular component is eliminated while preserving the tissue's physiological properties [4,12]. Decellularized tissues have the potential to be repopulated with specific cells, such as patient's cells to create a bespoke therapy [13].

In this review, we aimed to summarize 3D models of digestive system cancers including colorectal, gastric, and esophageal cancers, with particular emphasis on models that utilize decellularization techniques, which brings new potential to future investigations and a better understanding of the behavior of cancer cells.

## 2 Extracellular matrix (ECM)

ECMs are tissue components that regulate various cellular functions and act as scaffolds for cellular components [[14], [15], [16], [17]]. This 3D network consists of fibrillar and non-fibrillar molecules that interact with cell surface receptors, enabling signal transfer within cells [15,18]. The effects of ECM on cell function can be mediated in different ways. One way is through the direct binding of cell surface receptors or co-receptors. This can affect cell anchorage, mechanotransduction, and intracellular signaling pathways. Another way is through remodeling caused by aberrant presentation of growth factors and the actions of enzymes [16,19]. The characteristics of the matrix components divide ECM into two categories: interstitial and pericellular matrices. The pericellular matrix, also known as the basement membrane, contains Collagen IV, laminins, nidogens, heparan sulfate proteoglycans (PGs), perlecan, and agrin [15]. However, interstitial matrices provide structural support for cells and are rich in fibrillar collagens, proteoglycans, fibronectin, glycosaminoglycans (GAGs), and elastin [5,20]. This environment also includes growth factors (GFs), cytokines, and matrix-remodeling enzymes [15,21].

To coordinate various cellular functions, cells depend on extra-cellular receptors such as integrins [22], discoidin domain receptors (DDRs) [23], voltage-gated ion channels [15], cell-surface PGs [24], and CD44 [25]. Cell adhesion is primarily facilitated by integrins, which act as major receptors; as a result of their ability to bridge the actin cytoskeleton with ECM components and enhance outside-in signaling cascades, mostly through focal adhesion kinase (FAK) and Src tyrosine kinases; these signaling cascades result in many cellular functions [26,27]. Integrins play a crucial role in enabling cancer-associated fibroblasts (CAF) to carry out their function [26]. The activation of CAFs can be regulated by the expression of integrin α_v_β_6_ in cancer cells. When normal colonic fibroblasts are co-cultured with colorectal cancer cells, it has been observed that the levels of CAF markers (α-SMA and FAP) in the fibroblasts are directly related to the expression of integrin α_v_β_6_ in CRCs. Additionally, integrin α_v_β_6_ in CRCs plays a role in activating CAFs through TGF-β, and these activated CAFs, mediated by integrin 6, can enhance CRC cell invasion. Differences in gene expression of integrin α_11_, a receptor for fibrillar collagen, have been confirmed in CAFs compared to normal fibroblasts in the tumor stroma of non-small cell lung carcinoma (NSCLC). The tumorigenicity of NSCLC was found to increase when co-implanted with mouse embryonic fibroblasts (MEFs) expressing integrin α_11_. Recent data also shows that high expression of integrin α_11_ in CAFs, along with α-SMA, leads to collagen reorganization and tissue stiffness, promoting tumor growth and metastasis in NSCLC [28].

Additionally, the integrin content of subcellular structures acts as an ECM stiffness sensor, influencing matrix rigidity rate. ECM stiffness is a characteristic of tumors that differentiates them from normal tissues. It is related to a surge in the rate of metastasis and less positive results for patients. Additionally, it disrupts tissue morphogenesis by increasing cell tension. ECM stiffness is the aggregation of ECM proteins that enclose packs of hyaluronic acid (HA) gel-like structures. Integrin plays a crucial role in cancer progression by regulating cell behavior through signaling between the ECM and cellular cytoskeleton. Increasing ECM stiffness enhances integrin-dependent mechanotransduction, promoting malignancy. Inhibition of integrin signaling reduces invasion, while induced integrin clustering fosters focal adhesions, and increases phosphoinositide 3‐kinase (PI3K) signaling and invasion. Integrin-induced focal adhesions activate the ERK pathway, vital to cancer proliferation, survival, migration, and invasion [26,29,30].

DDR receptors are unique among receptor tyrosine kinases due to their distinct activation mechanism and their ability to bind to collagens and other ECM macromolecules. DDRs play a crucial role in regulating cellular functions like proliferation, differentiation, and migration. However, unregulated DDR activity can lead to diseases such as cancer and fibrosis. CD44 can act as a receptor (for GFs, cytokines, matrix metalloproteinases (MMPs), and hyaluronan), part-time PG, and controller of signal transduction. Different forms of CD44, including the standard type, play a role in the development of tumors and the ability of cancer stem cells (CSCs) to start the growth of tumors [15].

Recently, Extracellular vesicles (EVs), are referred to as lipid bilayer structures containing nucleic acids, lipids, proteins, and signaling molecules. They have been acknowledged as an essential element of ECM that is derived from the plasma membrane and can be divided into different categories: Exosomes (50–100 nm), microvesicles (100–1000 nm), and apoptotic bodies (1–5 mm) [15,31,32]. Exosomes contain biomolecules that can influence recipient cells and contribute to the renewal, restructuring, degradation, and mineralization of ECM [20]. The protein composition of exosomes includes membrane fusion proteins, proteins involved in the formation of EVs, heat shock proteins, myosin heavy chain class II proteins, integrins, and transmembrane proteins [32].

The major sources of ECM are stromal cells including pericytes and fibroblasts. Fibroblasts are present in a vast range of connective tissues such as the bone marrow, lymph nodes, ovaries, and solid tumors. Pericytes are found in a particular location which is around the endothelial cells of blood vessels [5].

Fibroblasts play a critical role in forming and renewing the ECM because they produce the macromolecules that make up its structure (like collagen, fibronectin, and laminin) and secrete enzymes that modify and degrade these macromolecules. Studies have shown that fibroblasts are cells with great flexibility and the ability to translate signals from various sources into modifications in the elements of ECM. Several signals, including cytokines, chemicals, and environmental signals such as heat and mechanical forces, can contribute to ECM makeover [5,33]. ECM remodeling occurs in pathological conditions like fibrosis and cancer, besides physiological conditions [[34], [35], [36], [37]].

## 3 The role of ECM in tumor progression

As tumor cells proliferate, there are notable modifications in the surrounding ECM [38,39]. These modifications involve unusual alterations in both the quantity and composition of ECM; including higher secretion of fibronectin and collagens [35,40]. The tumor microenvironment (TME) consists of all non-cancerous cells surrounding the tumor, such as fibroblasts, endothelial cells, neurons, adipocytes, and immune cells, as well as non-cellular elements like the extracellular matrix and various signaling molecules such as chemokines, cytokines, growth factors, and extracellular vesicles [41]. The stromal elements found in the TME may arise from the differentiation of existing fibroblasts, adipocytes, hematopoietic stem cells (HSC), pericytes, and epithelial cells undergoing epithelial-mesenchymal transition (EMT) [42,43].

EMT refers to a cellular process in which cells undergo a transformation, they lose polarity and epithelial cell-cell adhesion molecules (e.g., E-cadherin) while increasing mesenchymal markers (e.g., N-cadherin, fibronectin, and vimentin) [44,45]. The supportive cell components of the tumor are commonly known CAFs in modern times, which are the most abundant cell populations playing major roles in mediating an immunosuppressive TME [42,46]. Under the influence of tumor growth factor β (TGF‐β) and cyclooxygenase‐2 (COX‐2), fibroblasts are recruited to the tumor location and transformed into CAFs [26]. CAFs are in charge of ECM deposition during tumor progression; They can release different types of collagens, laminins, fibronectins, proteoglycans, periostin, and tenascin C that facilitate the progression of tumor and metastasis [42]. Single-cell RNA sequencing has led to the discovery of distinct CAF subpopulations amongst pancreas, breast, and colorectal cancers. In a recent study, Elyada et al. recognized a previously unknown subset of fibroblasts within pancreatic cancer, which they have named antigen-presenting CAFs (apCAFs). These apCAFs were found to coexist with the already-known myofibroblastic CAFs (myCAFs) and inflammatory CAFs (iCAFs). Researchers are working to identify specific subtypes of CAFs due to their complexity. Presently, while α-SMA + or FAP + CAFs are the two most major markers, the deletion of each phenotypic CAF yielded contrasting outcomes. Reduction of αSMA + cells during pancreatic ductal adenocarcinoma (PDAC) progression produced poorly distinct growths and decreased survival. Depletion of FAP + cells led to a boost in the population of antitumorigenic cytotoxic CD8^+^ T cells and a slowdown in the growth of pancreatic tumors. Vimentin, which plays a crucial role in the creation of the cytoskeletal network, is greatly presented in all types of fibroblasts. Consequently, this marker is extensively utilized to visually distinguish fibroblast populations in immunohistochemical and immunofluorescent studies. Vimentin is found in a variety of mesenchymal cells and in epithelial cells that are undergoing EMT. This makes it less specific as a marker for CAFs [47].

Tumor progression can be enhanced by an augmented accumulation of matrix proteins as they disrupt adhesion between cells and intensify the signaling of growth factors. However, studies have shown the reduction of fibrillar collagens may encourage the development of cancerous behavior [35,48,49]. Increased levels of glycosaminoglycan hyaluronic acid in the ECM leads to a higher risk of malignancy and poor prognosis. Hyaluronic acid serves as both a signal for inducing mesenchymal transition and a substrate for migration [35].

In addition to biochemical changes, ECM can undergo structural changes during the progression of tumors. Instead of relaxed and unorganized fibrils, collagen I are frequently aligned in a straight manner and positioned either parallel to the epithelium or extending perpendicularly into the tissue [40]. It is believed that the closely aligned collagen fibers serve as pathways for cancerous cells' migration out of the tumor [35]. Upon analysis of the collagen levels in cancer stroma and normal gastric mucosa, it was found that gastric cancer exhibited a substantial rise in collagen deposition and the fibers were tightly packed, resulting in a dense appearance [49].

Moreover, ECM's biomechanical characteristics change the state of the disease. For instance, the stroma of tumors is stiffer than normal stroma [40]. The excessive activities of LOX (lysyl oxidase) secreted by tumor cells can be considered as one of the reasons for increasing tissue stiffness, as LOX catalyzes collagen cross-linking and thus increases matrix stiffness [35,50,51]. According to Baker et al. the level of LOX was found to be significantly high in tumor tissues. This increase was even more substantial in metastatic tumors, indicating that LOX plays a crucial role in tumor progression [52]. In both lab experiments and animal models, it was observed that LOX stimulates the division of endothelial cells and promotes tumor angiogenesis by activating the Akt-VEGF pathway in colorectal cancer (CRC). Furthermore, intracellular LOX is strongly associated with reduced survival rates and serves as a predictive indicator for colon cancer metastasis to the lungs and liver [53]. Yet, Csiszar and colleagues showed that in individuals with CRC, there was a decrease in LOX mRNA expression, suggesting that LOX had a suppressive impact on tumors [54]. LOXL1 is essential in suppressing tumors by controlling cancer growth, invasion, and metastasis through the inhibition of yes-associated protein (YAP). On the other hand, LOXL2 is associated with the amount of colon cancer invasion and metastasis and can serve as an autonomous predictive biomarker. Additionally, reducing LOXL2 activity can slow down the spread of colon cancer cells, and lead to cell cycle arrest and apoptosis *in vitro* and *in vivo* [53].

Signals YAP1 can affect CAFs to promote matrix stiffness [26]. Studies have shown that increasing tissue stiffness can interfere with some in-and-out signaling pathways; for instance, the Rho-associated protein kinase myosin light chain (Rho-ROCK-MLC), which encourages an increased level of integrins, focal adhesion, cell contractility, and EMT markers, which they enhance the ability of cancer cells for metastasis [13,55].

The stiffness of ECM can hinder the efficient absorption or distribution of drugs to the intratumoral region [56]. This may occur due to an increased amount of collagen, which can bind to PGs and enhance the stability of ECM components. Studies have demonstrated that the administration of collagenase can enhance IgG diffusion to the tumor region. As tumors progress, they exhibit another trait known as ECM degradation [26]. Tumor and stromal cells exhibit elevated levels of proteases that break down the ECM components, affecting tumor progression in different ways. Initially, the gradual breakdown of healthy ECM is replaced by tumor-derived ECM as a result of proteolytic degradation. Second, the motility of cancer cells can be enhanced through the process of ECM degradation. Third, when soluble molecules like growth factors bind to ECM, they become inactive and insoluble. However, proteases can release them and activate their function. For instance, fibronectin has a strong affinity for insulin-like growth factor binding protein-3, FGF-2, and VEGF-A. Tumors with increased protease activity break down the extracellular matrix, releasing these growth factors and making them more available for use [57]. Finally, long ECM components can be cleaved to produce shorter fragments with unique functions that may have either pro- or anti-tumor properties when compared to original full-length ECM components. These fragments have structures similar to cytokines and chemokines, called matrilines, which play an important role in angiogenesis [58]. Different proteases can cleave ECM including MMPs [59].

Target-specific proteases including MMPs, can degrade ECM components, which is a crucial process for the regulation of ECM [58,60]. The MMP family consists of different types of MMPs, including collagenases, gelatinases, stromelysins, matrilysins, membrane-type MMPs, and other MMPs [61]. MMPs are key enzymes for ECM degradation which have an elevated level of activity in the state of diseases like cancer than normal conditions [62]. MMPs promote tumor invasion by degrading the basement membrane, allowing cells to invade surrounding tissue or blood vessels. Furthermore, MMPs cause the overexpression of several growth factors, including vascular endothelial growth factor (VEGF), fibroblast growth factor 2 (FGF-2), and TGF-β. This overexpression promotes cell proliferation and angiogenesis [63]. The connection between MMP-2, MMP-9, and tissue inhibitors of matrix metalloproteinases (TIMPs) in the invasion and spread of cancer cells is well understood. These proteins may serve as biomarkers since they can differentiate between CRC patients and healthy controls when found in serum and feces. Studies have shown that MMP-2 and MMP-9 biomarkers are more effective in diagnosing CRC than other biomarkers like CEA and CA19-9. However, using serum MMP-9 to diagnose advanced malignancies in the CRC family-risk population screening may not be beneficial. In a separate study, levels of MMP-9 were assessed in stool samples of 125 individuals with colon cancer, indicating its potential as a noninvasive indicator of CRC. Elevated levels of MMP-2 and MMP-9 in healthy tissue near colorectal tumors were discovered to be linked to a negative outcome for patients with colorectal cancer. Moreover, the regulation of MMP-9 by TIMP-2 is effective in predicting the prognosis of patients with CRC. Research also showed that TIMP-2 hinders the spread and invation of cells by controlling MMP-9 at the mRNA or protein level, potentially offering better prognostic value for colorectal cancer patients compared to TIMP-2 or MMP-9 individually [53].

MMP-2 interacts with integrin to promote the survival pathway of signal transducer and activator of transcription 3 (STAT3) in cells. STAT3 stimulates tumor angiogenesis and acts as a pathway for cancer cell survival [26]. Additionally, STAT3 is commonly acossiated with poor clinical outcomes. Increasing evidence suggests that overactive STAT3 can play a role in promoting immunosuppression in tumors through various mechanisms (including decreasing the expression of immune-stimulating factors including interferons (IFNs), pro-inflammatory cytokines (IL-12, TNF-α) and chemokines (CCL5, CXCL10), and increasing the expression of certain cytokines and growth factors (IL-6, IL-10, TGFβ, and VEGF)) [64]. In colon cancers, the expression of α_v_β_6_ integrin leads to the secretion of MMP-9 and activates the protein-kinase-C pathway. Further research has demonstrated that MMP-9 secretion triggered by α_v_β_6_ integrin has a vital role in the progression of colon cancer. Overexpression of MMP-9 has been linked to decreased survival rates as well as metastasis in both breast and colon cancers [65].

Angiogenesis is a vital process for tumor progression in which new blood vessels are formed from established vasculature because the rapid growth of cancerous cells cannot be sustained by the simple diffusion of oxygen alone [[66], [67], [68], [69]]. Proangiogenic factors play an important role in promoting the development of blood vessels that supply nutrients and oxygen to tumors, thereby facilitating their growth and progression. VEGF [70] and basic fibroblast growth factor (bFGF) [71] are two of the most vital and widely studied proangiogenic factors and their overexpression can enhance the development of tumors by stimulating angiogenesis [70,72]. VEGF-A is a crucial factor in regulating the growth and maintenance of abnormal blood vessels. It promotes the multiplication and survival of vascular endothelial cells and increases vascular permeability. VEGF-A interacts with VEGFR-1 and VEGFR-2 and their co-receptors NRP-1 and NRP-2 to exert its pro-angiogenic effects. Although VEGFR-1 has a higher binding affinity to VEGF-A, VEGFR-2 is more effective in promoting angiogenesis. Therefore, the VEGF-A/VEGFR-2 signaling pathway is considered the most critical mechanism in vessel formation. VEGF-B and placental growth factor (PGF) are members of the VEGF family that bind to VEGFR-1. VEGF-B has a weaker potential to induce angiogenesis in most tissues and acts more as a pro-survival factor rather than a pro-angiogenic one. On the other hand, PGF promotes stronger angiogenic responses in multiple tissues. This may be confusing since both growth factors interact with the same type of receptor. VEGF-C and VEGF-D predominantly induce lymphangiogenesis by binding to VEGFR-3 and have received less attention in tumor vessel formation compared to other VEGF family members [66]. FGFs are glycoproteins that signal by binding to cell surface heparan sulfate proteoglycans. They are liberated from the extracellular matrix through heparinases, proteases, or particular FGF-binding proteins. FGF signaling through FGFR regulates various biological functions involving tumor cells and the surrounding stroma, such as cellular proliferation, resistance to cell death, motility, invasiveness, metastasis, and angiogenesis. FGF-2, also called bFGF, is a crucial pro-angiogenic mediator that plays a significant role in both physiological conditions and tumor progression. It affects endothelial cells through a paracrine signaling mechanism and is released by tumor and stromal cells or mobilized from the extracellular matrix. FGF-2, in combination with VEGF, enhances angiogenesis by stimulating the release of MMPs, plasminogen activators, and collagenase that aid in breaking down and arranging the extracellular matrix. Recent research has found that FGF signaling plays a key role in regulating the development of blood and lymphatic vessels by influencing endothelial metabolism, which in turn affects MYC-driven glycolysis. This process is essential for activities like endothelial cell sprouting, movement, and growth. FGF activity has been linked to resistance against anti-angiogenic treatments in tumors. It is suggested that tumor cells may activate the pro-angiogenic FGF pathway as a way to evade VEGF-targeted therapies [73].

Chemokines that stimulate angiogenesis include CXCL8 (IL-8), CXCL1, CXCL2, CXCL3, CXCL5, CXCL6, and CXCL7. While some of these chemokines are known to interact with other receptors, particularly CXCR1, the key role of CXCR2 in mediating the angiogenesis induced by these chemokines has been demonstrated *in vivo* using CXCR2 gene-targeted animals. On the opposite side, there are angiostatic chemokines. CXCL9, CXCL10, and CXCL11, induced by IFNg, and CXCL4 which is not induced by IFNg. The three IFNg-inducible chemokines (CXC9-11) inhibit the growth of new blood vessels stimulated by VEGF or angiogenic CXC chemokines. IFNg and IL-12 are believed to inhibit angiogenesis using three angiostatic chemokines. It has been observed that the CXCR3 receptor mediates the activity of CXCL9, CXCL10, and CXCL11. CXCL4 is the first chemokine that was identified to have angiostatic activity. Previous studies have suggested that CXCL4 has diverse activities, including interfering with the growth factor interactions with GAGs, particularly heparan-sulfate proteoglycans. CXCL4 and CXCL10 have been observed to inhibit the interaction of bFGF and VEGF with their specific receptors by hindering the binding to heparan sulfate and subsequent dimerization [74].

**Fig. 1:**
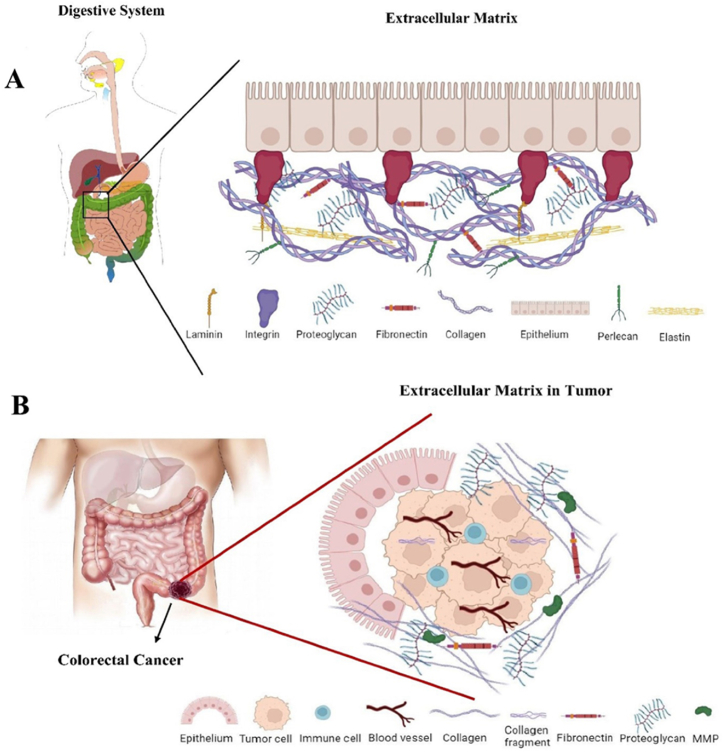
A) Schematic illustration of major components found in the heterogeneous ECM network of the digestive system. B) Remodeling of ECM during colorectal cancer progression. Abnormal makeup of ECM includes elevated levels of collagen, fibronectin, and matrix enzymes such as MMPs. Immune cells are frequently recruited to the tumor site in advanced-stage tumors, facilitating the progression of cancer. With the help of proangiogenic factors, new blood vessels are formed from previous vasculature. Collagen is frequently aligned in a straight manner and positioned either parallel to the epithelium or extending perpendicularly into the tissue which facilitates the migration of tumor cells. Additionally, an increased amount of collagen, which can bind to PGs can enhance the stability of ECM components which hinders the ability of drug absorption.

Angiogenesis is an intricate process that involves the proliferation and migration of endothelial cells and the breaking down of the basement membrane of blood vessels and associated ECM. After the proliferation of endothelial cells and the initial formation of tubes, the recently created vessels transform into arterioles and venules that are essential for supplying blood to tumors. ECM is composed of components promoting the process of tumor angiogenesis; including a diverse group of proteins such as laminin, fibronectin, proteolytic enzymes (MMPs), a disintegrin and metalloproteinases (ADAMs), and disintegrin and metalloproteinase with thrombospondin motifs (ADAMTs) [70,72]. Studies have shown that ADAM-17 is involved in the process of angiogenesis, as demonstrated in *in vivo* experiments on endothelial cells. Blocking ADAM-17 not only changed the shape of the endothelial cells but also reduced their growth while not affecting cell death. It was found that ADAM-17 affects angiogenesis by activating MMP-2 through VEGF. This was confirmed when endothelial cells without ADAM-17 did not show an increase in MMP-2 after being treated with VEGF. In contrast to ADAM-17, ADAMTS5 caused endothelial cell death even when VEGF was present, a powerful factor that promotes angiogenesis and growth. ADAMTS1 and ADAMTS8 have also been found to have the antiangiogenic domain (first thrombospondin repeat (TSR1)) from thrombospondin. Both of these proteins hindered endothelial cell growth and inhibited the vascularization induced by growth factors in different experiments. Laminin peptides from the α1 chain have been found to promote angiogenesis *in vivo*. The specific receptors responsible for laminin's pro-angiogenic effects are not yet fully understood, but it is suggested that the α6β1 receptor may be significant in the formation of blood vessels [72]. The necessity of α5β1 and finronectin in developmental angiogenesis is clear, but their role in tumor angiogenesis is still being studied. Previous research demonstrated that blocking the fibronectin-α5β1 interaction with antibody antagonists inhibited angiogenesis in the chick chorioallantoic membrane assay (CAM).(Fig. 1 A and B).

## 4 Drugs targeting ECM

Chemotherapies use a variety of methods to kill or slow the growth of cancer cells (Table 1). They can classified into different categories. For instance, Antimetabolites such as 5-fluorouracil (5-FU) and gemcitabine replace in RNA or DNA in place of their usual nucleotide counterparts. 5-FU disrupts DNA and RNA synthesis by incorporating into DNA, whereas gemcitabine causes DNA harm to hinder replication. Yet, the effectiveness of chemotherapy hinges significantly on internal mechanisms of resistance to chemotherapy that increase DNA repair, drug removal, prosurvival signaling, and halting the cell cycle [41].

**Table 1:** Drugs targeting ECM components under clinical trials.

Drug	Trial ID	Target/Mechanism	indication	phase	Ref.
SAR439459	NCT04729725	TGFβ	Advanced Malignant Solid	1	[75]
			Neoplasm/Metastatic Malignant		
			Solid Neoplasm/Unresectable		
			Malignant Solid Neoplasm		
	NCT04643002		Plasma Cell Myeloma Refractory	1/2	[75]
	NCT04524871		Advanced Liver Cancers	1/2	[75]
NIS793	NCT04390763	TGFβ	Metastatic Pancreatic Ductal	2/3	[75]
	NCT04935359		Adenocarcinoma		
ABBV151	NCT03821935	GARP- TGF-β1	Advanced Solid Tumors Cancer	1	[75]
AVID200	NCT03834662	TGFβ	Malignant Solid Tumor	1	[75]
Simtuzumab	NCT01472198	LOXL2	Pancreatic cancer	2	[76]
Cilengitide	NCT00689221	Integrin	Glioblastoma	3	[76]
Dacarbazine	NCT00066196	Integrin	Metastatic melanoma	2	[76]
ATN-161	NCT04177108	Integrin	Malignant glioma	1/2	[76]
Dasatinib	NCT00826449Dasatinib	Tyrosine kinases	Lung	1/2	[77]
TG-0054 (burixafor)	NCT02104427	CXC4R	Multiple myeloma, lymphoma	2	[77]
Calcipotriol Paricalcitol	NCT03596073	Vitamin D analogue	Early-stage skin cancer, breast cancer, pancreatic cancer	1/2	[41]
	NCT04617067				
	NCT02030860				
	NCT03138720				
	NCT04054362				
Reparixin, AZD5069, SX-682	NCT02001974	CXCR1/2 antagonist	Metastatic breast cancer, Colon cancer, Prostate cancer	1/2	[41]
	NCT02370238				
	NCT02499328				
	NCT04599140				

Recently, numerous clinical trials have confirmed the healing impact of TGF-β-targeted medications on various tumor and fibrotic conditions. TGF-β signaling is vital for embryonic development and adult homeostasis, but its dysregulation is significantly linked to fibrosis and tumorigenesis. The process is intricate, with TGF-β acting as a tumor suppressor in early cancer cells and a tumor enhancer in advanced cancer cells. It controls multiple functions such as EMT, ECM buildup, immune infiltration, and CAF activation [75]. Previous research has shown that the rigid and cross-linked ECM not only promotes tumor growth but also hinders the ability of immune cells and cancer-fighting drugs to target cancerous cells. As a result, targeting ECM stiffness has emerged as a potential strategy to treat cancer and overcome drug resistance. Several druggable regulators of ECM stiffness, including mechanosensors and mechanotransducers, have been identified. Fibrillar collagen, a major contributor to increased ECM stiffness, can be directly targeted by recombinant collagenase to deplete it. Additionally, inhibitors of LOX, LOXL2, LOXL3, and integrin have been developed and have shown anticancer activities in preclinical studies [76].

## 5 Decellularization definition, methods, and characterization

### 5.1 Decellularization definition

The creation of bioscaffolds derived from extracellular matrix has been greatly sought after for use in tissue engineering and regenerative medicine [78,79]. The extracellular matrix is obtained from a tissue by the process of decellularization [80,81]. The main objective of decellularization is to eliminate the native cells and genetic components, such as DNA, from the ECM while preserving its structural, biochemical, and biomechanical cues. The suggested guidelines for evaluating the effectiveness of eliminating these elements are as follows: the ECM has been decellularized needs to have: (1) less than 50 ng double-stranded DNA (dsDNA) per mg ECM dryweight, (2) less than 200 bp DNA fragment length, and (3) no visible nuclear material by 4′,6-diamidino-2-phenylindole (DAPI) staining [80].

CDM (cell-derived matrices) and dECM (decellularized extracellular matrices) are types of ECM processed with similar decellularization methods. While CDM is obtained from lab-grown cells or tissues, dECM is derived from real tissues. CDMs can be easily obtained through a non-invasive process, offering advantages such as the availability of target tissue-specific cell lines, and minimal risk of pathogen transfer. Each cell could produce a unique ECM that may have a distinct immunomodulatory effect and tissue regeneration capability [82]. The composition and properties of CDM can be altered by modifying intrinsic factors (cell choice and genetic engineering) and extrinsic factors (additives and culture conditions) [83].

Cell-derived matrices are typically produced by treating stromal cells, such as fibroblasts, with ascorbic acid to enhance matrix production. However, ascorbic acid toxicity limits its use for generating matrices from cells. An alternative method is the technique of macromolecular crowding (MMC)**.** Louisthelmy et al**.** demonstrated that MMC culture is a useful technique for generating cancer cell-derived matrices. The addition of inert macromolecules to the cell culture medium helps mimic the crowded environment of living organisms by creating steric hindrance and electrostatic repulsion. This technique restricts the diffusion of cell-derived molecules such as procollagen and proteinases, which accelerates the deposition of ECM [84].

dECM scaffolds serve as storage units for bioactive substances and interactions between cells and the matrix. providing some advantages. One of the benefits is that dECM holds definite tissue-specific memory, which can drive tissue-specific differentiation due to the presence of memory factors and cues found in the native ECM. Moreover, dECM retains many features of the complex ECM design and internal structure, such as pore shape and collagen fiber arrangement, which play a crucial role in controlling cell attachment, growth, and differentiation. Additionally, their mechanical characteristics make them ideal for tissue repair and regrowth [85].

The chemical composition of CDM may closely resemble that of dECM from the same tissue source, but it does not possess the structural or mechanical characteristics of native tissue, thus dECM is the most effective method for preserving the complex range of proteins, GAGs, PGs, and other matrix components present in native tissue [83,86,87] (Table 2).

**Table 2:** Comparison between dECM and CDM.

	Advantages	Disadvantages
dECM	The same chemical and physical properties as the original tissue	Scarcity of animal/donor tissue
		Batch-to-batch variability
		Depend mainly on animal/human donor
CDM	Tunable constitution for proposed applications	Achieving native tissue structure is difficult
	Constant quality	Less characterized for different tissue types
	Maintainable, reduction of animal usage	Growth and expansion difficulties

### 5.2 Decellularization methods

**Fig. 2:**
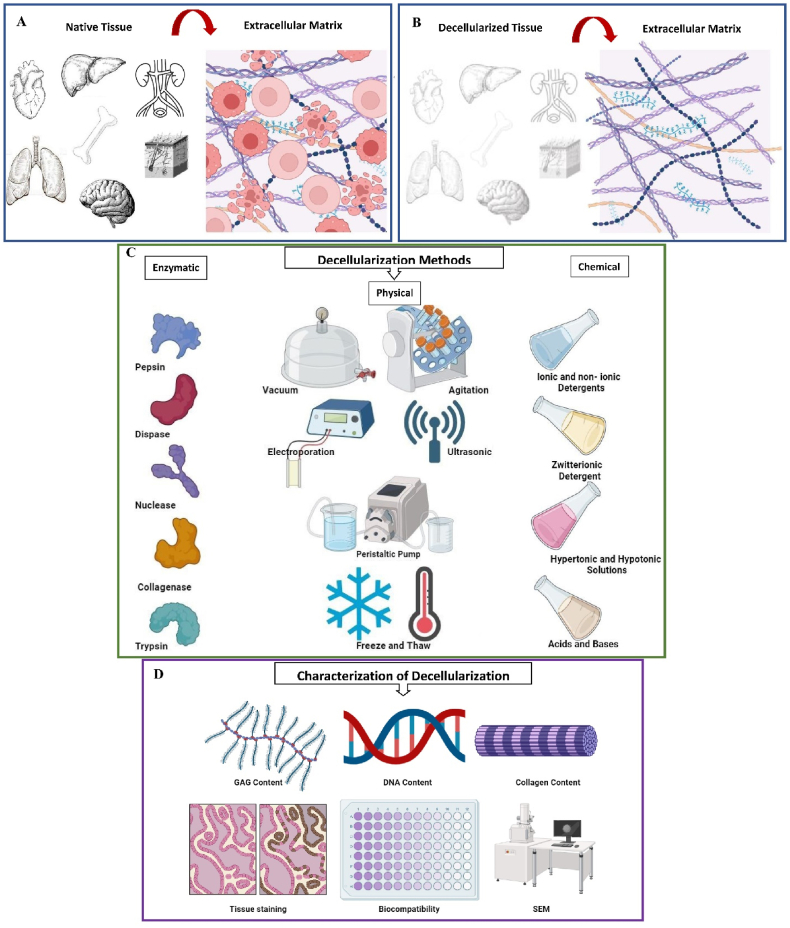
Process of engineering tissues through the utilization of decellularized ECM. A,B), Through the decellularization procedure, the native cells and genetic components are removed from the ECM, such as DNA, while characteristics of native tissues are preserved, C), The process of decellularization can be achieved through enzymatic, physical, or chemical methods, D), A proper characterization method is needed to confirm if the desired decellularization process is done. Common characterization techniques include extracellular and cytoplasmic components assays, cell residual assays, and observation of the general microscopic structure of decellularized ECM.

ECM of native tissue contains cells, but in decellularized tissue, cells are removed from this matrix (Fig. 2. A and B). Numerous techniques for decellularization have been suggested (Fig. 2. C). Although decellularization can be achieved through chemical (sodium dodecyl sulfate (SDS) and Triton X-100), enzymatic (DNase, EDTA (Ethylenediaminetetraacetic acid) and Trypsin), or physical (Freeze-Thaw and High Hydrostatic Pressure or HHP) methods, each approach has its advantages and disadvantages [80]. The complexity of tissues makes the decellularization process highly variable, resulting in inconsistent outcomes. Therefore, decellularization is typically accomplished through a combination of these methods to overcome disadvantages [88,89]. Table 3 summarizes the features of common Decellularization methods.

**Table 3:** Summary of commonly used decellularization methods [80,88].

Category	method	Description	advantage	disadvantage
*chemical*	SDS	An ionic surfactant that is widely utilized	Effective elimination of cellular constituents	The microstructure of ECM is damaged(collagen and GAGs)
				Hard to remove from tissues when washing
	Triton X-100	A nonionic surfactant that is often utilized	Complete removal of DNA in combination with ammonium hydroxide	Good preservation of tissue's ultrastructure and mechanical characterization
*enzymatic*	DNase	Used to break down DNA	Not harming the ECM	Hard to remove from tissues when washing
		Fragments	Highly effective in removing DNA waste	Only functions when used alongside a treatment that interferes with cell membrane
	EDTA	A Chelating agent, often used with trypsin	Causing disruptions between cells and ECM	Not efficient on its own
			Reducing salt- and acid-soluble proteins	
	Trypsin	An enzyme that is frequently utilized in combination with EDTA	Effective removal of cell surface	Extended periods of exposure can damage ECM
			Disrupting adhesion of cells to matrix	
*physical*	Freeze-thaw	Requires switching between −80° and normal biological temperatures of around 37°	Preservation of both biochemical elements and biomechanical characteristics	Remaining DNA fragments
	High hydrostatic pressure (HHP)	Requires pressures exceeding 60Mpa	Enhancing the level of chemical contact and eliminating waste materials in tissues	ECM integrity can be affected by elevated pressures
				Remaining DNA fragments

Detergents, including both ionic and non-ionic types, have the ability to interfere with the interactions between hydrophobic and hydrophilic molecules. They can eliminate immunogenic cellular elements by breaking down cell membranes and separating DNA from proteins. However, potent detergents may negatively impact the ECM by interfering with protein elements. Ionic detergent is a successful chemical method for decellularization, breaking down nuclear and cytoplasmic membranes by interfering with various interactions such as lipid-lipid, lipid-protein, DNA-protein, and protein-protein interactions. This process results in the removal of cells and genetic material. SDS, sodium deoxycholate (SDC), sodium lauryl sulfate, sodium lauryl glutamate, sodium lauryl ester sulfate, and Triton X- 200 are the most common ionic detergents. It has been confirmed that SDS treatment can completely eliminate both the original cells and genetic materials. The level of SDS concentration is crucial in the process of decellularization. Increased SDS concentration reduces DNA content and scaffold strength in dECM, whereas lower concentration enhances collagen retention, minimizes ECM protein denaturation, but raises cytotoxicity and cellular remnants. Ionic detergents negatively affect the structure of the ECM by disrupting protein-protein interactions. Moreover, removing SDS is challenging because of its strong bonds with ECM proteins, which could potentially harm recellularized cells. Non-ionic detergents are gentler than ionic detergents when it comes to solubilizing cell membranes and separating DNA from proteins by disrupting lipid-lipid, lipid-protein, and DNA-protein interactions, while preserving protein-protein interactions. Non-ionic detergents have the ability to remove cellular materials without harming collagen's arrangement due to their inability to alter protein structures. However, due to their slighter effects, they are incapable of removing nuclei and DNA. As a result, these detergents always need help from other solutions or physical methods to make sure all cellular components are fully removed [85].

Enzymatic treatments target specific chains within cellular fragments or cell-matrix adhesions to effectively remove cell components and unwanted ECM constituents with precision. DNase, a common type of nuclease, is often utilized for decellularization because of its ability to selectively remove DNA while preserving proteins by breaking down nucleic acid sequences. Following the application of substances that enhance inner gaps and openness, DNase is typically employed to facilitate faster and more efficient penetration into tissues. As well as assisting in the washing out of detergents, the infiltrated DNase has the ability to eliminate remaining genetic material and cellular debris. DNase in combination with detergents is necessary for effectively removing cellular materials, as it can achieve over 95 % removal of DNA. However, the extended processing time required for nucleases can negatively impact the structure and mechanical stability of ECM, leading to a decrease in ECM components like GAG, laminin, and collagen IV. Another frequently used enzyme for decellularization procedures is Trypsin. It breaks the peptide links on the carbon end of arginine and lysine, leading to the detachment of cellular elements from ECM. Trypsin has been commonly utilized for its ability to efficiently remove cells from the ECM without causing harm to them. However, trypsin's protein-cleaving properties may potentially harm the ECM and affect its mechanical strength. It is recommended to use appropriate concentrations and exposure durations when using trypsin [85].

The breakdown of ECM elements increases worries about the harmfulness of the chemical substances employed during the decellularization procedure. Consequently, new methods are emerging that eliminate the need for potent chemicals by utilizing physical principles to disrupt cells and remove cell-matrix binding proteins, necessitating a rinsing process to remove cellular debris. The most popular physical methods include freeze-thawing and high hydrostatic pressure. The freeze-thaw process includes alternating between freezing temperatures around −80 °C and biological temperatures around 37 °C for a specific number of cycles. However, the protocols can be customized by adjusting the temperature difference and/or the number of freeze-thaw cycles. Freeze-thaw alone is not a sufficient method for removing cells and genetic material, so it is typically used in conjunction with other techniques and substances. The HHP method works by applying pressures above 600 MPa to disrupt the cellular membrane. Unlike the freeze-thaw process, high hydrostatic pressure has the potential to be utilized independently. Hashimoto et al. used high hydrostatic pressure to decellularise porcine corneas. The corneas were treated at a pressure of 980 MPa, and staining with Hematoxylin and Eosin (H&E) showed successful removal of cells [90].

Every tissue related to the digestive system is decellularized by different methods. And we have discussed the advantages and disadvantages related to that method. So far, the method of governor that does not have disadvantages in general and only includes advantages has not been introduced, and the research in this field continues.

### 5.3 Characterization methods

Since the removal process of cells can alter the makeup, physical characteristics, and strength of the ECM, it's important to have a proper characterization method to confirm if the desired decellularization process is done (Fig. 2. D) [89,91]. It is necessary to assess the impact of processing on the final product's composition and biological properties. The process of decellularization is considered successful if two conditions are met: complete removal of cellular material and preservation of matrix functionality. Although decellularization techniques cannot entirely remove cellular material, it is possible to quantitatively assay cell components such as dsDNA, mitochondria, or membrane-associated molecules like phospholipids [17].

Common histological staining and immunofluorescence methods are employed to confirm the effective elimination of cellular components through qualitative verification. The first step of assessment frequently involves the use of H&E, Hematoxylin is commonly utilized to verify the degree of decellularization whereas Eosin is used to detect non-nucleic components [91]. Measurement of remaining DNA can be performed by using commercial kits, polymerase chain reaction (PCR), fluorescent 4′,6-diamidino-2-phenylindole (DAPI), or nucleus staining methods (hematoxylin and Hoechst 33258) [89,92].

After completing the verification of cellular removal, understanding the effects of decellularization on the remaining ECM scaffold's mechanical and material properties is of interest. The presence of certain components after decellularization is crucial for dECM's functionality [17]. These components include collagen, elastin, laminins, fibronectin, and GAGs. Several histological techniques have been used for the compositional characterization of decellularized ECM, including Alcian blue staining for detecting GAGs, and Sirius red, Azan stainings and Masson's trichrome for detecting collagens. Besides these histochemical methods, immunochemical methods with specific antibodies are commonly employed to identify proteins. Lectins are often used to identify particular GAGs [89,93].

Dye-labeling techniques are commonly used for quantifying ECM components. Collagen, elastin, and GAGs can be accurately and quickly analyzed using these methods. Quantification of collagen can be achieved through the use of Picrosirius Red and 4-(Dimethylamino) benzaldehyde (DMAB), which detects hydroxyproline residues. Elastin quantification uses 5,10,15,20-tetraphenyl-21H,23H-porphine tetra-sulfonate (TPPS) dye, a synthetic porphyrin that is soluble in water, which attaches to the basic and hydrophobic amino acids. Glycosaminoglycans, such as chondroitin sulfate, keratan sulfate, dermatan sulfate, and heparan sulfate, can be quantified by using a cationic dye, such as 1,9-dimethyl methylene blue (DMMB) or toluidine blue, which interacts with the negatively charged sulfate groups through an ionic interaction. Colorimetric assays are fast but have limitations like matrix interference, low specificity, and limits of detection. These constraints can be surpassed by supplementing methods [93]. Additionally, mass spectrometry and gel electrophoresis can be utilized for compositional analysis of decellularized ECM [89]. Recent studies reported that peptides that can hybridize with denatured collagen triple helix and denatured collagens can be visualized with these peptides [89].

The rheological method can be used to determine the physical properties of ECM, while its thermal stability can be evaluated using differential scanning calorimetry. The study of rheology entails investigating how substances respond to various mechanical pressures and comprehending how their physical characteristics influence their flow patterns. Rheology evaluates several important properties of materials, one of which is their viscoelastic character. Elasticity pertains to a material's capacity to revert to its initial form following deformation, whereas viscosity relates to the capability to stream and disperse energy. The extracellular matrix displays both elastic and viscous characteristics due to proteins like collagen and elastin that can stretch and ease. Another crucial rheological property of the ECM is its stiffness or rigidity, which influence cellular behavior and tissue function. Rigidity is influenced by the makeup and arrangement of the ECM's elements; for instance, collagen contributes to a more rigid nature, whereas an increased amount of PGs can enhance compressibility and pliancy [93].

Differential Scanning Calorimetry (DSC) is a thermal analysis technique employed to assess the resilience of biomolecules like proteins in their original state. DSC gauges the heat transfer linked to alterations prompted by temperature shifts that align with protein thermal destabilization or “heat-induced unfolding”. DSC measures the temperature difference between an empty reference cell and a sample cell containing the sample, which provides information on protein stability. The thermal absorption from the sample chamber is gauged as it demands additional energy to achieve an equivalent temperature to the reference chamber. A protein is considered more stable if it has a higher thermal transition temperature [93]. Analysis of the structure of decellularized ECM can be accomplished by using scanning electron microscopy (SEM) and transmission electron microscopy (TEM) [89]. The use of TEM is a fundamental method for obtaining a better understanding of tissue including the order, diameter, and spacing of collagen fibers. SEM provides reduced clarity compared to TEM and is primarily employed to analyze the structural shape of tissues [91].

The functional biological assessment of the ECM-based structure is conducted using *in vitro* and *in vivo* tests. Evaluating the biodegradability of ECM-derived substances can forecast their duration in a living setting. Enzymes or enzyme-free physiological media may be used to monitor changes in mass or structural integrity over time [93,94]. In vitro assays provide information on cell viability, morphology, proliferation, and differentiation. Cultured cells can be placed on ECM-derived materials, and various methods including Live/Dead staining, cell counting, and MTT assay can assess the fundamental cellular reaction in relation to metabolic function, survival, and growth while engaging with the ECM substances. The MTT (3-(4,5-dimethylthiazol-2-yl)-2,5-diphenyltetrazolium bromide) reduction assay is an effective method used to measure metabolic activity in living cells. The test relies on MTT reduction by active cells to produce purple formazan crystals. The active mitochondria's succinate dehydrogenase system causes this conversion. However, it has recently been proposed that NADH is the principal reducing agent responsible for MTT reduction, generated via the metabolic processes of glycolysis and oxidative phosphorylation [95].

**Fig. 3:**
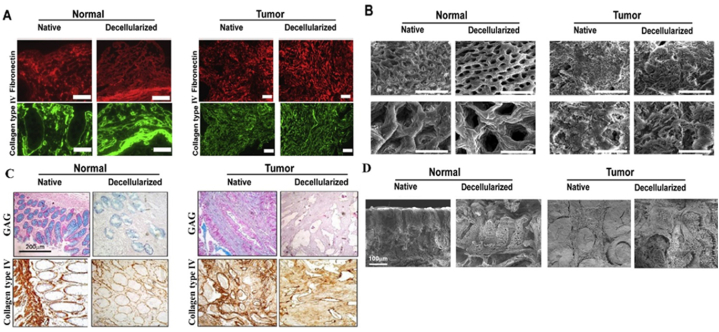
Decellularized normal and tumor human colorectal tissue, A [96] and C [97]) Immunohistochemical expression of fibronectin, collagens type IV, Glycosaminoglycans(GAGs) in native and decellularized normal and tumor sections, B [96] and D [97]) Scanning Electron Microscopy (SEM) of native and decellularized normal and tumor tissue. A decrease in total proteins was observed in decellularized samples compared to native samples. The native matrix shows a homogenous surface in the SEM images, where colonic crypts are visible and the ultrastructure is preserved to a large extent in the decellularized samples.

However, *in vivo* testing is necessary to fully understand the interaction between ECM-derived materials and living tissues*. In vivo* tests, in this scenario, usually entail implantation studies in animal models. This model requires careful consideration of the research objectives, selecting a suitable animal model, ethical and regulatory guidelines, as well as the target tissue or organ. After implantation, the surrounding tissues are examined to determine the host's immune response to the ECM-derived biomaterial. This analysis helps to evaluate the biocompatibility and integration of the biomaterial with the host's tissue. To further assess the regeneration potential and functionality, other techniques such as immunohistochemistry are used to observe the formation of new tissue, angiogenesis, and morphology [93] (Fig. 3 A-D).

## 6 Application of decellularization tissue in digestive system cancers

### 6.1 Colorectal cancer

In recent years, there have been numerous attempts to create new models that can replicate the intricate connections within the TME, with a particular focus on accurately representing the complex biochemical and biomolecular aspects of the tumor ECM. The colorectal cancer ECM is profoundly remodeled and disorganized and constitutes a key component that influences cancer trademarks, such as cell differentiation, multiplication, angiogenesis, invasion, and metastasis [4]. The use of 2D plastic cell culture promotes the growth of specialized cells that are actively dividing, but it can not accurately replicate the diverse environment found in TME containing cell populations with varying proliferative, stem-like, chemo-resistant, pluripotent, and metastatic capabilities [98].

Furthermore, the process of angiogenesis, cannot be reproduced in 2D models [99]. Previous attempts to reproduce the natural 3D cellular environment have relied on protein gels and scaffolds that only partially imitate the complex nature of native tissues and lack architectural support and signaling proteins provided by ECM which may affect cell behavior and progression of cancer [[99], [100], [101]]. Numerous reports have detailed fascinating approaches that involve utilizing decellularized ECM from natural tissues, in which cellular components are eliminated while preserving the tissue's physiology [4].

Tissues from cells, animals, humans, or patients, can be decellularized and used as a scaffold for 3D cell cultures. It has also been used in bioprinting methods to produce flexible 3D structures and as a constituent in both synthetic and natural cell culture systems, offering adjustable constructs with bioactive ECM components [4,85]. For instance, decellularized porcine jejunum derived from the patented Biological Vascularized Scaffold (DE:302014007893; Bio-VaSc) has been utilized as a platform to generate a 3D colorectal cancer tissue model. This scaffold is defined as a small intestinal submucosa with preserved mucosa (SISmuc) which preserves the mucosal tissue layer, including the crypt, villi, and basement membrane structures, allowing us to study cancer cell growth, EMT process, cell invasion across the basement membrane, and metastization. In this model, two different human colon cancer cell lines (Caco2 and SW480) were monocultured or cocultured with fibroblasts. Both cell lines showed a decreased number of proliferating cells compared to 2D cultures, showing a more accurate correlation with primary colon cancer in every stage. Furthermore, the treatment using 5-FU demonstrated effectiveness only in 3D coculture [102].

According to another *in vitro* 3D colon cancer model presented by Alabi et al. intact wild type (WT) and colon cancer susceptible decellularized mouse colons (DMC) utilized, ECM structural proteins, and the mechanical properties, including stiffness of native tissue were preserved. The human colon cancer cell line (HT-29) was able to proliferate and differentiate due to permissive conditions provided by DMC. It was observed that the ability of cells to differentiate in DMC was greater than Matrigel ™ (a mouse tumor cell line-derived extracellular matrix extract). Moreover, based on invasion tests, DMC derived from polyps in a mouse model prone to colon cancer boosted the invasion and infiltration of cancer cells, as well as the immortalization of non-cancerous cells when contrasted with DMC from WT mice [100].

Romero-López et al. discovered that tumor ECM plays a crucial role in enhancing the tumor's vascularity and altering the metabolism of both the tumor and blood vessels by extracting and comparing ECM from normal human colon and colon tumors that had metastasized to the liver. They observed that the vessels developed in tumor ECM imitated the vasculature of tumors, as they exhibited similar characteristics, such as varying vessel diameters within the network and fractal dimension, a numerical gauge that measures the complexity of a network. Moreover, the protein composition of both ECMs was determined; although normal and tumor ECMs have proteins in common, tumor ECM contains additional components. Collagen I was present similarly in each sample, but collagen IV, VI, and XIV, Fibrillin, Emilin, Vitronectin, and Laminin (γ1) were increased in tumor ECM. Also, several ECM proteins that were only present in tumors were identified, including Fibronectin, Periostin, Versican, Thrombospondin-2, and Tenascin. To determine the metabolic state of the cells, they examined the free/bound ratios of NADH in both tumor and endothelial cells. It was found that similar to *in vivo* findings, cells seeded in tumor ECM had greater levels of free NADH, indicating a higher rate of glycolysis, compared to those seeded in normal ECM [99].

Parkinson et al. used patient-derived scaffolds (PDS), obtained by decellularization of surgically resected tumor material, as a growth substrate for colon cancer cell line HT29 to represent the heterogeneous nature of colorectal cancer. After 3 weeks of PDS culture, they found that HT29 cells varied their gene and protein expression profiles noticeably compared to 2D-grown HT29 cells. While markers associated with pluripotency were increased, markers associated with proliferation were consistently decreased in PDS-grown cells compared to those grown in a 2D environment. When comparing the changes induced by PDS in HT-29 cells with relevant clinical information from individual patients, they found a significant correlation between stemness/pluripotency markers and tumor location, as well as between EMT markers and cancer mortality [98].

In another study presented by Genovese et al. a procedure for the purification of tissue-derived ECM using mucosae from healthy human colon, perilesional area, and colorectal cancer was developed to assess the role of ECM in the modification of tissue homeostasis and tumorigenesis. The positioning and arrangement of seeded monocytes and cancer cells in the initial tissue were distinctly controlled by the three varieties of ECM. Despite perilesional and CRC-derived ECM, healthy ECM constrained the invasion of cancer cells. Furthermore, the different types of ECM showed distinct levels of support for cell proliferation, with the tumor-derived ECM demonstrating the highest effectiveness, followed by the perilesional-derived ECM, and lastly the healthy-derived ECM [103]. Piccoli and colleagues developed an organotypic 3D-bioactive model by decellularizing human biopsies. It was found that 5 days after recellularization with HT-29 cells, the 3D tumor matrices induced an over-expression of *IL-8*, a pathway mediated by DEFA3 and a chemokine that is required for the growth and spread of cancer [104].

### 6.2 Esophageal cancer

Esophageal cancer ranks as the sixth most prevalent form of cancer worldwide and is particularly lethal because of the absence of early detection techniques and successful treatment options. The majority of research on esophageal cancer characteristics is typically conducted using either 2D well plates or xenograft animal models [105]. Recent tissue engineering methods are also being utilized to build esophageal cancer models to create artificial tumor tissue, which includes growing cancer cells in synthetic polymer scaffolds, such as poly (lactic-*co*-glycolic acid) (PLGA) and poly (lactic acid) (PLA) scaffolds or natural collagen/matrigel to artificially make tumor tissues, which ECM of the human esophagus is not accurately represented by them [106].

Formerly, studies have explored the use of decellularized rat esophagus for potential medical purposes in humans. Since the rat esophagus does not contain submucosal glands, any effort to replicate the human esophagus in a rat model would lack certain important anatomical characteristics present in humans. To address the issue, Chaitin and colleagues prepared a porcine esophageal decellularized esophageal matrix (DEM) to study cancer cell growth. To observe their growth, human esophageal squamous cell carcinoma (ESCC) and human primary esophageal fibroblast cells (FBCs) were cultured in the DEM. The expression of periostin, a protein that supports cell adhesion on the matrix, was detectable when cells were cocultured since the periostin expression of KYSE30 on the DEM was stimulated by stromal fibroblasts [106].

Brennan et al. developed a 3D esophageal tumor tissue model using a biomimetic decellularized esophageal matrix in a customized bioreactor. Setting up this system for perfusing culture media for cells was a straightforward process that ensured that cells receive a continuous and dynamic supply of nutrients for growth and eliminate waste effectively. To set up The expression of cancer-related markers of KYSE30 cells was analyzed and compared with formalin-fixed, paraffin-embedded (FFPE) ESCC tissue biospecimens. KYSE30 cells seeded in the DEM in the dynamic bioreactor expressed different cancer marker expressions than those in the static well plate while having similarities with the FFPE-ESCC biospecimens [105].

### 6.3 Gastric cancer

Gastric cancer ranks as the fourth most prevalent form of cancer globally and the second leading cause of death associated with cancer. Even though surgical therapy and chemotherapy lead to enhanced therapeutic outcomes, survival rates are still unsatisfactory due to the tumors’ high drug resistance. Since the development of gastric cancer is influenced by the tumor microenvironment, creating a biologically relevant microenvironment is becoming more crucial in *in vitro* studies. Kim et al. presented a new bioink that combines gastric dECM and cellulose nanoparticles (CN) to create a mechanically reinforced substance that could mimic the biochemical microenvironment found in gastric cancer. They found that CN can improve the mechanical properties of the matrix including stiffness, which can support the progression of gastric cancer since it has been speculated that cancer cells' behavior is regulated by ECM stiffness. It was confirmed that gastric cancer cells formed larger aggregates due to an increase in the stiffness of ECM [107] (Table 4).

**Table 4:** Tissue origins, decellularization agents, and cell lines used in the production of digestive system cancer models.

origin	source	Decellularization method	Cell line	Drug	Ref.
Animal-derived	SIS muc (small intestine submucosa + mucosa of decellularized porcine jejunum)	4%SDS	Caco2, SW480	5-FU	[4,102]
		DNase I			
	Wild type (WT) and colon cancer susceptible mouse colons (DMC)	4 % SD	HT-29	–	[100]
		2000 kU DNase I			
	Rat liver and lung tissues	1 % SDC	HT-29, Caco2 and SW480	5-FU, Irinotecan alone, Irinotecan + 5-FU, Oxaliplatin alone, and Oxaliplatin + 5-FU	[108]
		Phospholipase			
	Porcine Esophagus	1%Triton X-100, ammonium hydroxide, trypsin, EDTA, DNase I, RNase, SD, EDTA, dimethyl sulfoxide (DMSO)	KYSE30	–	[106]
	Porcine Esophagus	0.2 % Triton X-100 freeze/thaw	KYSE30	–	[105]
		DNA lysate			
	Porcine gastric tissue	1 % SDS	AGS, SNU-1, KATO3,	5-FU	[107]
		1 % Triton X-100			
Human-derived	Colorectal cancer tissue	0.1 % SDS	HT-29	**-**	[98]
		EDTA			
	Healthy colon mucosa, primary CRC, healthy liver, and CRLM	**-**	HT-29, HCT-116	5-FU and 5-FU combined with Irinotecan (FOLFIRI)	[109]
	Healthy colon submucosa, liver metastasis	2 % SDC	SW620, SW480, HCT116	–	[99]
		1 % Triton-X100 for colon tissue,			
		1 % SDS			
		1 % Triton-X100 DNAse for liver tissue			
	Healthy mucosa (N) and tumor lesion (T) from CRC patients	4 % SDC	HT-29, HCT-116	5FU, FOLFIRI	[110]
		DNase-I			
	Mucosae from healthy colon, perilesional area, and colorectal carcinoma	5 mM EDTA 10 % DMSO	CD68^+^,HT-29, LoVo, SW480	–	[103]
		1 % Triton X-100			
		10 mM sodium cholate hydrate			
		50 mM Tris-HCl			
		Centrifugal rotation			
		Ionic and nonionic surfactants			
		Mechanical mixing			
	Normal mucosa and cancer lesion tissue samples	–	HT-29	–	[104]

## 7 Application of 3D model in cancer study

### 7.1 Metastasis

The use of decellularized tissues has also been employed in the examination of the metastatic process of CRC [4]. Tian and colleagues developed a cancer metastasis model by seeding CRC cells in a biomatrix developed by decellularizing mouse lung and liver ECM. In this model, the spontaneous development of 3D cell colonies histologically, molecularly, and phenotypically mimicked *in vivo* metastases. Following the injection into mice, the metastases derived from culturing CRC cells on decellularized liver and lung scaffolds maintained their tissue-specific tropism. They also found that the engineered metastases contained signet ring cells, an *in vivo* pathological discovery that has not been documented in *ex vivo* model systems. Moreover, it was demonstrated that *in vitro* acellular microenvironment can affect the sensitivity of CRC cell lines to chemotherapy since engineered Caco2 lung metastases exhibit greater responsiveness to chemotherapy interventions compared to engineered liver metastases of Caco2 [108].

In another study, D'Angelo et al. generated a 3D representation of CRC and compared it to cancer liver metastasis (CRLM) by utilizing decellularized ECM scaffolds derived from patients and seeded with the HT-29 CRC cell line. An increase in the capability of HT-29 cell proliferation and migration was demonstrated when cultured in cancer-derived scaffolds compared to healthy colon and liver tissues of the same patients. HT-29 cells grown in CLRM scaffolds showed signs of undergoing EMT, with a decrease in E-cadherin levels and an increase in vimentin expression. Studies were conducted on the result of 5-FU and FOLFIRI therapy on colorectal cancer cells grown in 3D patient-derived structures. The patient-derived scaffolds decreased the cellular response to drugs due to the intricate 3D environment they provided [109].

### 7.2 Drug assessment

Preclinical assessment of cytotoxicity, efficacy, and efficiency of drugs holds significant importance in the process of drug discovery. According to estimates, just 5 % of drugs that show activity in 2D cellular models are effective in clinical trials [110]. Due to this rationale, researchers were compelled to create more advanced *in vitro* methods, that is, 3D models, which mimic specific features of solid tumor tissues [111].

Sensi et al. suggested a 3D preclinical model, derived from the patient, which could imitate the patient's disease *in vitro* for drug assessment purposed by seeding HT29 and HCT116 cell lines in decellularized CRC tissue and healthy colon mucosa near that. After five days of recellularization with HT29 and HCT116 cell lines, the 3D model of colorectal cancer exhibited reduced responsiveness to 5-fluorouracil (5FU) therapies in contrast to 2D cultures. According to xenograft experiments, the half-maximal inhibitory concentration (IC_50_) of 5FU was similar to that calculated in the 3D model [110]. In another study, Hoshiba focused on the role of EMT in the contribution of ECM remodeling to the acquisition of 5FU resistance. It was confirmed that ECM remodeling during tumor progression leads to increased CS chains to promote EMT and ABCB1 upregulation via TGF-β signaling, which contributes to the acquisition of chemoresistance [44].

## 8 Limitations, advantages, and challenges

Preclinical investigations into cancer characteristics and drug resistance are typically carried out using either 2D *in vitro* cultures of cancer cells or xenograft animal models. However, 2D models can't correctly mimic *in vivo* environments, owing to the lack of a 3D architecture for appropriate cell-matrix interactions. Therefore, they often fail to sufficiently model normal tissue and disease processes. As previously mentioned, a process that removes cellular components from tissues without or minimally affecting ECM structure and composition is decellularization, which shows great potential in obtaining matrices derived from biological sources. This technical advancement enabled the creation of 3D culture models of tumor cells that preserved ECM and tissue structure, offering a promising method for generating more significant cancer models. Additionally, the presence of main structural proteins and soluble factors in the decellularized scaffold is the major benefit of biological-derived matrices instead of synthetic polymers for 3D tumor engineering *in vitro* [104].

Since decellularized ECMs can either be derived from animals or humans, studies have shown some animal-derived matrices are not able to represent human tissues because ECM proteins exhibit a high degree of conservation among different species [4,100,103]. Recent research has focused on patient-derived scaffolds to study tumor-stroma interactions [4]. Patient-derived 3D models are beneficial platforms for supporting the growth of cancer cells, improving the ability of cancer cells to migrate, triggering genetic and protein responses in malignant cells that correspond to particular clinical evidence about the patient's disease, allowing for a better understanding of the diversity of cancer among different patients [98,110].

In addition, this model has the potential to offer vital predictive information regarding the efficacy of cancer treatments, which can then aid in making clinical decisions tailored to individual patients [98]. One of the major weaknesses of patient-derived scaffolds is the restricted quantity of tumor tissue available from each person. This is because the tissue is derived from biopsies or surgical resections, from which most tissue is necessary for additional diagnostic molecular and histological analysis. Another important consideration is that the normal mucosa next to the tumor, which is often regarded as a healthy control from the same person, actually represents a middle state between normal and tumor tissue [4].

When aiming to study tumor cell–stroma interactions, the lack of multiple cell populations is one of the major drawbacks [108,109]. Future research may include resident cell types to better recreate the environment of the tissues. This will enable us to study the interactive communication between cells and their surrounding environment [108].

## 9 Conclusion

The primary benefit of decellularized matrices is their remarkable resemblance to the original tissues. In addition, decellularized tissues can be utilized to create cancer models by repopulating them with various cell lines. This technique is highly advantageous in the field of research as it allows for the study of cancer without the need for live subjects. The process also enables the creation of customized cancer models that can be tailored to specific research needs. Overall, the use of decellularized tissues in cancer research is an important and rapidly advancing area of study. By utilizing these models, we can thoroughly examine the interactions within the TME and accurately assess the effectiveness of various cancer treatments.

## Data availability statement

All data are fully available without restriction.

## CRediT authorship contribution statement

**Zahra Seifi:** Writing – original draft, Investigation, Conceptualization. **Mozafar Khazaei:** Writing – review & editing, Writing – original draft. **Danial Cheraghali:** Writing – original draft, Investigation. **Leila Rezakhani:** Writing – original draft, Supervision, Project administration.

## Declaration of competing interest

The authors declare the following financial interests/personal relationships which may be considered as potential competing interests: Dr. Leila Rezakhani reports was provided by Department of Tissue Engineering, School of Medicine, Kermanshah University of Medical Sciences, Kermanshah, Iran. If there are other authors, they declare that they have no known competing financial interests or personal relationships that could have appeared to influence the work reported in this paper.
